# VO_2_ Kinetics and Metabolic Contributions Whilst Swimming at 95, 100, and 105% of the Velocity at VO_2 max_

**DOI:** 10.1155/2014/675363

**Published:** 2014-06-18

**Authors:** Ana C. Sousa, João P. Vilas-Boas, Ricardo J. Fernandes

**Affiliations:** ^1^Centre of Research, Education, Innovation and Intervention in Sport, Faculty of Sport, University of Porto, Rua Dr. Plácido Costa, 91, 4200-450 Porto, Portugal; ^2^Porto Biomechanics Laboratory, LABIOMEP, University of Porto, Rua Dr. Plácido Costa, 91, 4200-450 Porto, Portugal

## Abstract

A bioenergetical analysis of swimming at intensities near competitive distances is inexistent. It was aimed to compare the transient VO_2_ kinetics responses and metabolic contributions whilst swimming at different velocities around VO_2max⁡_. 12 trained male swimmers performed (i) an incremental protocol to determine the velocity at VO_2max⁡_ (vVO_2max⁡_) and (ii) three square wave exercises from rest to 95, 100, and 105% of vVO_2max⁡_. VO_2_ was directly measured using a telemetric portable gas analyser and its kinetics analysed through a double-exponential model. Metabolic contributions were assessed through the sum of three energy components. No differences were observed in the fast component response (*τ*_1_—15, 18, and 16 s, *A*_1_—36, 34, and 37 mL · kg^−1^ · min⁡^−1^, and Gain—32, 29, and 30 mL · min⁡^−1^ at 95, 100, and 105% of the vVO_2max⁡_, resp.) but A2 was higher in 95 and 100% compared to 105% intensity (480.76 ± 247.01, 452.18 ± 217.04, and 147.04 ± 60.40 mL · min⁡^−1^, resp.). The aerobic energy contribution increased with the time sustained (83 ± 5, 74 ± 6, and 59 ± 7% for 95, 100, and 105%, resp.). The adjustment of the cardiovascular and/or pulmonary systems that determine O_2_ delivery and diffusion to the exercising muscles did not change with changing intensity, with the exception of VO_2_ slow component kinetics metabolic profiles.

## 1. Introduction

In the 1920s a sustained period of research in human exercise physiology emerged, with swimming (along with cycling and running) as one of the primary areas of research in sport sciences. In this sport, the maximal oxygen uptake (VO_2max⁡_) reveals itself as an important physiological parameter, expressing the swimmers' maximal metabolic aerobic performance [1, 2] that is one of the primary areas of interest in training and performance diagnosis [3]. In fact, determining the VO_2max⁡_ is the main aim of some studies, but the capacity to sustain this intensity in time is a recent topic of research. A big temporal gap exists between the pioneer study where VO_2max⁡_ was measured in swimming [4] and the first studies where time to exhaustion at the velocity corresponding to VO_2max⁡_ (Tlim-vVO_2max⁡_) was assessed [5–7]. Though being conducted in nonspecific swimming pool conditions (swimming flume), these studies evidenced that Tlim-vVO_2max⁡_ depended inversely on the vVO_2max⁡_ but was not related with VO_2max⁡_. To date, few studies have been conducted in conventional competition conditions [1, 8–13], and none has proposed how VO_2_ kinetics might impact concerning distinct vVO_2max⁡_ swimming intensities.

The quantification of the dynamic characteristics of VO_2_ kinetics has gained popularity in exercise physiology as a mean to unveil the mechanisms underlying the control O_2_ muscular consumption in humans during exercise [14]. Traditionally, the dynamic VO_2_ response to exercise has been studied at three intensity domains: moderate—below the anaerobic threshold, heavy—above the anaerobic threshold, and below the critical power and severe—above the critical power until the VO_2max⁡_ boundary [15, 16]. In the severe intensity domain, neither VO_2_ nor blood lactate concentrations ([La^−^]) can be stabilized, rising inexorably until fatigue ensues, with VO_2_ achieving its maximum value [17]. Here, the VO_2_ slow component is more pronounced compared to during heavy exercise, with its magnitude dependent on the duration and type of exercise [18]. More recently, the extreme exercise domain has been proposed for power outputs that lead to exhaustion before VO_2max⁡_ is attained [19], with the kinetics of VO_2_ characterized by the development of an evident fast component with insufficient time for the appearance of a discernible VO_2_ slow component [20]. Although the characteristics of the VO_2_ kinetics in the moderate and heavy intensity domains are well described (particularly in cycle and running ergometer exercise), few studies have investigated it during swimming around the VO_2max⁡_ intensity [7, 8, 11]. Moreover, these studies have only conducted a simplistic VO_2_ kinetics characterization by presenting the VO_2_ slow component as the unique kinetics parameter.

Given the current level of interest in exercise tolerance and VO_2_ kinetics, it is surprising that no study has examined both Tlim-vVO_2max⁡_ and VO_2_ kinetics response at intensities close to VO_2max⁡_. Trying to overcome this absence of data and aiming to disseminate knowledge to other forms of human locomotion, the purpose of this study is to compare the transient VO_2_ kinetics responses whilst swimming until exhaustion at different velocities around the VO_2max⁡_ intensity. In addition, the different metabolic contributions of each exhaustion exercise will also be analyzed. It was hypothesized that 5% of variability in swimming velocity will not promote significant changes in the VO_2_ fast component kinetics response phase but will promote distinct VO_2_ kinetics in the slow component phase.

## 2. Material and Methods

### 2.1. Subjects

Twelve well trained male swimmers (mean ± SD; age: 18.2 ± 4.1 yr, height: 179.4 ± 6.5 cm, body mass: 70.5 ± 5.8 kg, and mean performance for long course 200 m freestyle of 86.5 ± 3.7% of the 2013 world record for this event), participants in national level competitions, volunteered to participate. All the swimmers, specialized in middle distance freestyle events, trained at least eight times per week and competed in National Championships for at least 4 years. Their maturational index (according to Tanner scale) corresponded to the 4 or 5 stages. Swimmers were familiar with the testing procedures as they were involved in previous physiological evaluations. All subjects avoided strenuous exercise in the 24 h before each testing session and were well hydrated and abstained from food, caffeine, and alcohol 3 h before testing. The protocols were conducted at the same time of the day for each subject and were separated by, at least, 24 h. All participants (or parent/guardians when subjects were under 18 yrs) provided informed written consent before data collection.

### 2.2. Experimental Design

Subjects visited the swimming pool facilities on four different occasions over a two-week period. In the first session, VO_2max⁡_ and vVO_2max⁡_ were determined through an intermittent incremental protocol until exhaustion. In the subsequent visits, subjects completed three square wave exercises from rest to distinct percentages of vVO_2max⁡_ intensity until exhaustion to assess its time sustained. Encouragement was given to motivate the swimmers to perform their best effort in the incremental protocol and to perform as long as possible during the square wave exercises.

### 2.3. Incremental Protocol

Briefly, each subject performed an individualized intermittent incremental protocol for front crawl vVO_2max⁡_ assessment, with increments of 0.05 m · s^−1^ and 30 s intervals between each 200 m stage until exhaustion [9, 21]. According to these studies, the initial velocity was established according to the individual level of fitness and was set at the swimmer's individual performance on the 400 m front crawl minus seven increments of velocity. The velocity was controlled at each stage by a visual pacer with flashing lights in the bottom of the pool (TAR.1.1, GBK-electronics, Aveiro, Portugal). VO_2max⁡_ was considered to be reached according to primary and secondary criteria [22] and all ventilatory parameters mean values were measured over the last 60 s of the exercise. If a plateau less than 2.1 mL · min⁡^−1^ · kg^−1^ could not be observed, vVO_2max⁡_ was calculated as previously described [23].

### 2.4. Time to Exhaustion Tests

A total of three experimental exhaustive conditions were conducted in randomized order: 95, 100, and 105% of vVO_2max⁡_. Each square wave exercise consisted of three distinct phases: 10 min warm-up at 50% of the VO_2max⁡_, a short rest period (300 s), and the maintenance of the specific swimming intensity until exhaustion [8, 11]. The square wave exercises ended when the swimmers could no longer maintain the required velocity dictated by the visual feedback. Ventilatory parameters were calculated as the average values measured over the last 60 s of each exhaustive exercise.

### 2.5. Experimental Measurements

Respiratory and pulmonary gas-exchange variables were directly measured using a telemetric portable gas analyzer (K4b^2^, Cosmed, Rome, Italy), suspended over the water (at a 2 m height) in a steel cable following the swimmer along the pool and minimizing disturbances of the normal swimming movements. This apparatus was connected to the swimmer by a low hydrodynamic resistance respiratory snorkel and valve system (Aquatrainer, Cosmed, Rome, Italy [24]), which presents inspiratory and expiratory tubes of 86 cm length counting a volume of 847 mL from the mouthpiece to the turbine. It contains a dead space at the valves assembly, of 11.3 mL. In-water starts and open turns, without underwater gliding, were used. Heart rate (HR) was monitored and registered continuously by a Polar Vantage NV (Polar electro Oy, Kempele, Finland) that telemetrically emitted the data to the K4b^2^ portable unit. The gas analysers were calibrated before each test with gases of known concentration (16% O_2_ and 5% CO_2_) and the turbine volume transducer was calibrated by using a 3 L syringe. Capillary blood samples (25 *μ*L) for [La^−^] were collected from the earlobe during the 30 s intervals (incremental protocol) and immediately at the end of exercise during the 1st, 3rd, 5th, and 7th min of the recovery period, until maximal values were reached ([La^−^]_max⁡_), in both incremental and square wave exercises (Lactate Pro, Arkay, Inc., Kyoto, Japan).

### 2.6. Data Analysis

Firstly, occasional VO_2_ breath values were omitted from the analysis by including only those in-between VO_2_  mean ± 4 standard deviations. After verification of the data, individual breath-by-breath VO_2_ responses were smoothed by using a 3-breath moving average and time average of 5 s [21]. For VO_2_ kinetics analysis, the first 20 s of data after the onset of exercise (cardiodynamic phase) were not considered for model analysis. For this, a double-exponential (1) equation was used where a nonlinear least squares method was implemented in the MatLab Software to fit the VO_2_ data with the model:

(1) V˙O2(t)=A0+A1∗(1−exp⁡−(t−TD1/τ1))⁡ +A2∗(1−exp⁡−(t−TD2/τ2)⁡),

where VO_2_(*t*) represents the relative VO_2_ at the time *t*, *A*_0_ is the VO_2_ at rest (mL · kg^−1^ · min⁡^−1^⁡) and *A*_1_ and *A*_2_ (mL · kg^−1^ · min⁡^−1^), TD_1_ and TD_2_  (s), and *τ*_1_ and *τ*_2_  (s) are the amplitudes, the corresponding time delays, and time constants of the fast and slow VO_2_ components, respectively. The oxygen deficit (DefO_2_) was calculated as the multiplication of *A*_1_ and *τ*_1_. In turn, *A*_1_ was used to determine the gain (*A*_1_/velocity) of the primary fast component. The relative contribution of the slow component to the overall increase in VO_2_ at the end exercise was calculated as [*A*_2_/(*A*_1_ + *A*_2_)]. For the primary fast component to be accurately described by an exponential model, the duration of the VO_2_ primary rise before the onset of the VO_2_ slow component must be sufficiently long (≥4 times *τ*_1_), in all subjects at all swimming intensities.

The maximal metabolic expenditure (*E*_max⁡_) amounted during the square wave exercises was assumed to be the sum of the three components: aerobic (Aer), anaerobic lactic (Ana_lac_), and anaerobic alactic (Ana_alac_) energies [25–27]. The Aer contribution was calculated from the time integral of the net VO_2_ versus time relationship in the appropriate time ranges (mL O_2_) and then expressed in kJ assuming an energy equivalent of 20.9 kJ · lO_2_^−1^ [28]. The Ana_lac_ was estimated through the energy derived from lactic acid production (2):

(2) $$\begin{array}{c} \text{An}{\text{a}}_{\text{lac}}=b\cdot {\left[\text{La}\right]}_{\text{bnet}}\cdot M, \end{array}$$

where [La]_bnet_ is the net accumulation of lactate after exercise, *b* is the energy equivalent for lactate accumulation in blood (2.7 mL  O_2_  mM^−1^ · kg^−1^ [29]), and *M* (kg) is the mass of the swimmers (mL O_2_). Then, it was expressed in kJ assuming an energy equivalent of 20.9 kJ · lO_2_^−1^ [28].

The Ana_alac_ was assessed from the maximal PCr splitting in the contracting muscle:

(3) $$\begin{array}{c} \text{An}{\text{a}}_{\text{alac}}=\text{PCr}\cdot \left(1-e^{-t/\tau }\right)M, \end{array}$$

where Ana_alac_ is the anaerobic alactic contribution, *t* is the exercise time, *τ* is time constant of the PCr splitting at the onset of exhausting exercise (23.4 s [30]), *M* is the body mass, and PCr is the phosphocreatine concentration at rest. This latter was estimated assuming that, in transition from rest to exhaustion, its concentration decreases by 18.55 m-mole · kg^−1^ muscle wet weight (in a maximally working muscle mass equal to 30% of the overall body mass). Ana_alac_ was, thus, expressed in kJ by assuming an energy equivalent of 0.468 kJ · mole^−1^ and a P/O_2_ ratio of 6.25 [31].

### 2.7. Statistical Analysis

Individual, mean, and standard deviations (SD) values were used for descriptive analysis for all studied variables and measures of skewness, kurtosis, and the Shapiro-Wilk test allowed to assess the normality and homogeneity of the data. The differences in ventilatory, metabolic, and VO_2_ kinetics parameters and time sustained between the square wave exercises at 95, 100, and 105% of vVO_2max⁡_ were tested for statistical significance using ANOVA for repeated measures. When a significant *F* value was achieved, the Bonferroni post hoc procedures were conducted to locate the pairwise differences between the averages. Simple linear regression and Pearson's correlation coefficients were also used. All statistical procedures were conducted with SPSS 10.05 and the significance level was set at 5%.

## 3. Results

Time to exhaustion decreased with increasing swimming velocity: 344.09 ± 63.64, 194.17 ± 47.79 and 122.64 ± 20.06 s at 1.34 ± 0.05, 1.39 ± 0.06 and 1.46 ± 0.07 m · s^−1^ (corresponding to 95, 100 and 105% of the vVO_2max⁡_, resp.).  Table 1 lists the mean ± SD data regarding the ventilatory and metabolic parameters assessed during the incremental and the square wave bouts performed at different percentages of vVO_2max⁡_.

There were no differences between the determined physiologic parameters obtained in the incremental protocol and the square wave exercises and between the different time to exhaustion tests.  Table 2 shows the VO_2_ kinetics parameters obtained at 95, 100, and 105% of vVO_2max⁡_ during the square wave exercises.

**Figure 1:**
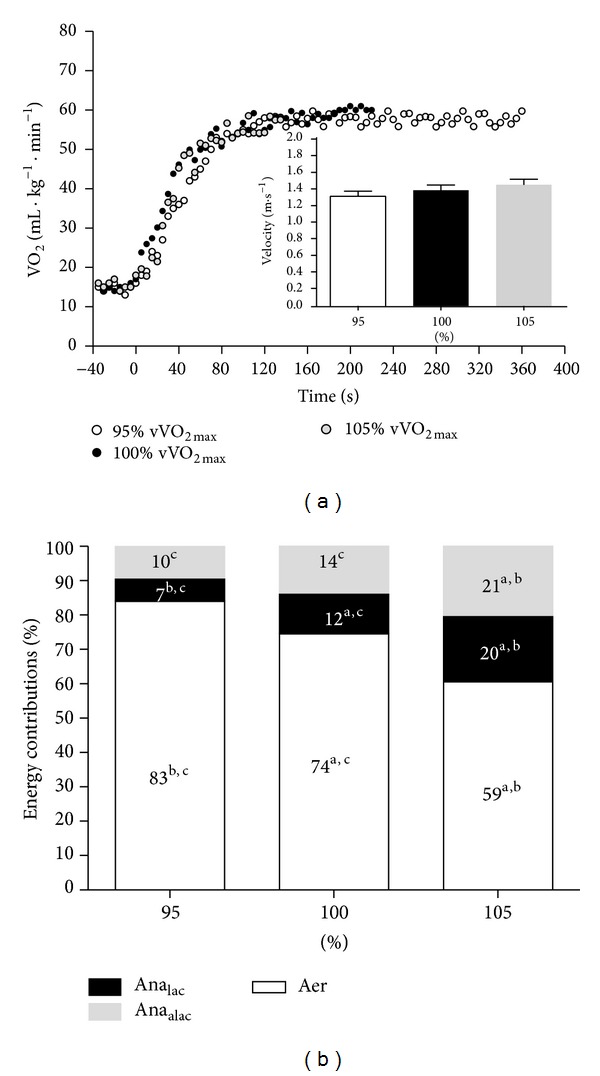
(a) VO_2_ kinetics individual response 5 s time average at 95, 100, and 105% of vVO_2max⁡_. The* insets* represent the mean velocity values of each time to exhaustion bout; (b) mean aerobic, anaerobic lactic, and anaerobic alactic perceptual contributions obtained during the square wave exercises at 95, 100, and 105% of vVO_2max⁡_ (differences between intensities are identified by a, b, and c for 95, 100, and 105% of vVO_2max⁡_, resp.; *P* ≤ 0.05).

At all intensities, the best data fit was obtained when the model incorporated a slow component (*R* ^2^ = 0.94, 0.92, and 0.90 for 95,  100, and 105%  of  vVO_2max⁡_ intensity) as opposed to a single exponential model, once a significant decrease in the sum of squared residuals occurred (the criterion measure used for each model). In fact, the *F*-test values evidenced a high heterogeneity between both models variances, also confirmed by the differences between their mean values. No differences were found between different time to exhaustion bouts regarding the fast component phase, but *A*_2_ was higher at 95 and 100% compared to the 105% vVO_2max⁡_ intensity and with physiological meaning (≥200 mL · min⁡^−1^) only at former intensities. In addition, the relative contribution of *A*_2_ to total VO_2_ kinetics response was similar in-between 95 and 100% and higher than 105% vVO_2max⁡_. Moreover, the TD_2_ was higher at 95% compared to 100 and 105% of vVO_2max⁡_ and *τ*_2_ was higher in 95 and 100% compared to 105% of vVO_2max⁡_. An individual VO_2_ kinetics response and the mean and SD values for the swimming velocity and the metabolic contributions at 95, 100, and 105% of vVO_2max⁡_ are shown in Figures 1(a) and 1(b).

Each square wave started with a sudden and exponential VO_2_ increase, independently of the swimming intensity. Differences between the Ana, An_alac_, and Ana_alac_ contributions were found between time to exhaustion intensities, with exception to the Ana_alac_ that was similar between 95 and 100% of vVO_2max⁡_.

**Figure 2:**
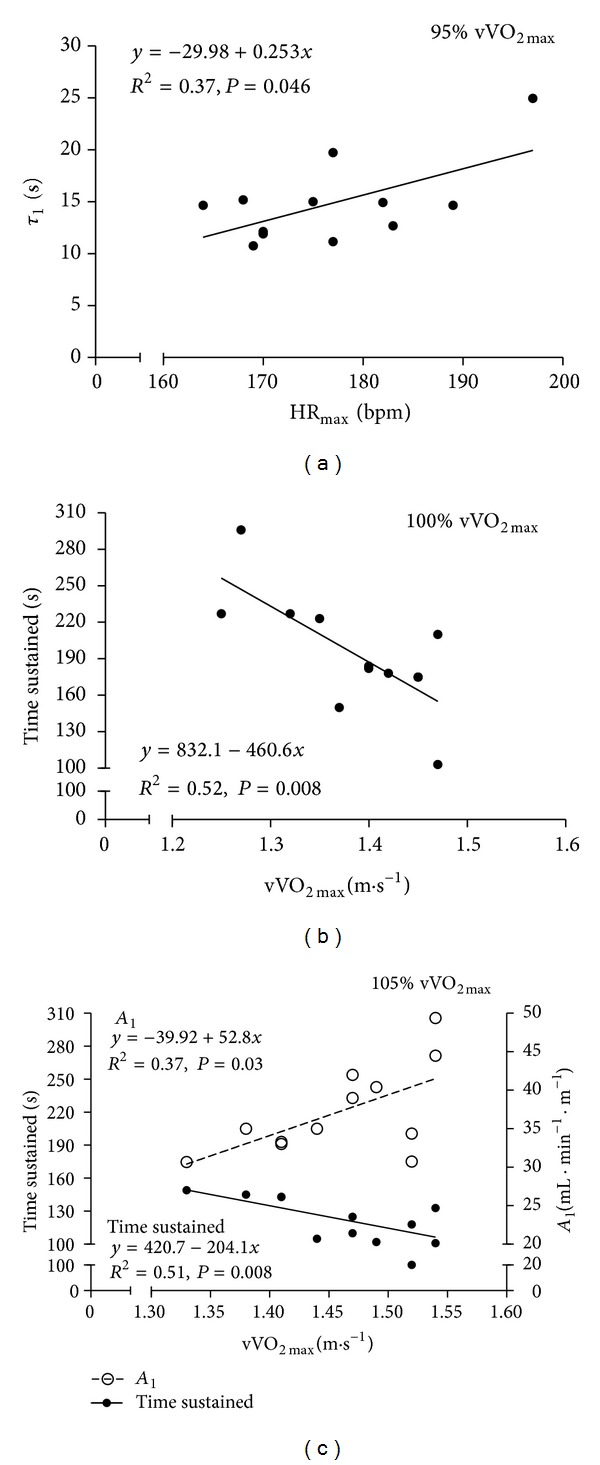
Relationships between maximum peak heart rate (HR_max⁡_) and fast component time constant (*τ*_1_) at 95% of vVO_2max⁡_ intensity (a), velocity and time sustained at 100% of vVO_2max⁡_ intensity (b), and velocity and time sustained (filled circles) and velocity and fast component amplitude (unfilled circles) at 105% of vVO_2max⁡_ intensity (c). The regression equations, determination coefficients, and significance level values are also identified.

In Figure 2 it is possible to observe the relationships between swimming performance indicators and VO_2_ kinetics parameters obtained at the different time to exhaustion bouts, with significant correlation values found between (i) HR_max⁡_ and *τ*_1_ at 95% of vVO_2max⁡_ (a); (ii) vVO_2max⁡_ and time sustained at 100% of vVO_2max⁡_ (b); and (iii) vVO_2max⁡_ and time sustained at 105% of vVO_2max⁡_, and, vVO_2max⁡_ and *A*_1_ (c).

## 4. Discussion

Studies investigating VO_2_ kinetics when performing to exhaustion have been conducted mainly in cycle ergometry and treadmill exercise, presenting a pretty simplistic approach when comparing different exercise intensities. The current study is the first attempt to examine and compare the VO_2_ kinetics during swimming to exhaustion at different velocities around the VO_2max⁡_ intensity. The exercise duration decreased when intensity increased, similarly to what was proposed for other cyclic sports. In addition, no differences were found in the VO_2_ fast component related parameters (*τ*_1_, *A*_1_, and Gain) between 95, 100, and 105% of vVO_2max⁡_, supporting our hypothesis that a 5% change in swimming velocity surrounding the VO_2max⁡_ intensity would not be sufficient to promote changes in the primary phase of VO_2_ kinetics response. However, *A*_2_ was higher at 95 and 100% compared to 105% of vVO_2max⁡_ corroborating the hypothesis that different swimming intensities near vVO_2max⁡_ would promote distinct VO_2_ slow component kinetic profiles. In addition, *E*_max⁡_ was different between the studied intensities.

VO_2max⁡_ is the most commonly measured parameter in applied physiological sciences. The mean values obtained at the end of the incremental protocol are in accordance with those presented for middle distance swimmers [5, 6], but lower than those described for elite swimmers [8–11], runners [32, 33], cyclists [34, 35], and rowers [36, 37], probably explained by the use of a larger muscle mass in these sports. Also, the observed [La^−^]_max⁡_ mean values are lower compared to other exercise modes, which can explain the lower *R* mean values found. This fact suggests that a lower metabolic acidosis occurs in swimming compared to other sports or that swimmers are less sensitive to it [5]. Furthermore, no differences were observed in the ventilatory and metabolic parameters between the incremental protocol and the square wave exercises, in agreement with the literature for 100% vVO_2max⁡_ swimming exercise [1, 5, 11]. In-between intensities comparison did not evidence changes in ventilatory and metabolic mean values, conversely to the differences found between 100 and 105% of vVO_2max⁡_ in running [32].

Specifically in swimming, only Demarie et al. [7] analysed a time to exhaustion at intensities different from 100% of vVO_2max⁡_, showing that swimmers were able to sustain ~375 s at 96% of vVO_2max⁡_, studying agreement with our current data (~344 s). The values reported in the current study for 100% vVO_2max⁡_ are also similar to those presented for highly trained swimmers performing at the same intensity [9–11] but are lower than others obtained in nonreal competition conditions [5, 6]. In addition, Alberty et al. [12, 13] conducted studies performed at 95 and 100% of the velocity of the 400 m front crawl (not measuring ventilatory parameters), observing a longer time to exhaustion compared to ours (~670 and 238 s). Collectively, these studies seem to evidence that time sustained at intensities around vVO_2max⁡_ depends also on the conditions in which they occurred. In fact, it has been reported that swimming flume might influence the VO_2max⁡_, vVO_2max⁡_, and the Tlim-vVO_2max⁡_ assessment, as well as the swimming technique, and, therefore, could explain the differences found [8].

Concerning the VO_2_ kinetics, the observed *τ*_1_ mean value is lower than values obtained for to 100 and 400 m front crawl all-out efforts [38, 39] but higher compared to the 200 m front crawl all-out efforts [2, 40]. Conversely, the value found is in accordance with previous reports for 7 min swimming at heavy [41, 42] and severe intensities [42] and for 400 m performed at 100% vVO_2max⁡_ [43]. The VO_2_ kinetics response was also characterized by a similar *τ*_1_ in-between the time to exhaustion tests, in line with previous cycling and treadmill ergometer studies who have showed that it remains constant as exercise intensity increases from moderate to heavy and to severe intensity domains, despite the increasing acidosis [44–47]. The current study also corroborates the absence of differences in *τ*_1_ between intensities around VO_2max⁡_ cycling exercise (90, 100, and 120%, [47]). It has been suggested that the characteristics of VO_2_ kinetics provide insights into the physiological mechanisms responsible for the control of, and the limitations to, VO_2_ kinetics following the onset of exercise [48]. Thus, similar *τ*_1_ values observed seem to suggest that an O_2_ delivery and diffusion are not influenced by a 5% external arousal in swimming at vVO_2max⁡_ intensity. In fact, at 95% of vVO_2max⁡_, a positive correlation was observed between HR_max⁡_ and *τ*_1_, evidencing that if a limiting factor exists, it may be related to peripheral factors (from convective O_2_ transport, to its diffusion and utilization in the muscles) and not to central ones (O_2_ delivery and transportation to the working muscles).

Complementarily, the *A*_1_ mean value obtained in this study is in accordance with previous reports for swimming at heavy intensity [41] but lower compared to higher intensities [2, 40, 42]. The similar *A*_1_ values across conditions do not corroborate the fact that an increase in amplitude is linearly related to the increase in exercise intensity [45–47]. However, when comparing intensities around VO_2max⁡_ in cycling exercise [47], differences have only been observed between 90% and 110% of vVO_2max⁡_. These results suggest that the 5% velocity changes on our experimental set were not sufficient to induce modifications in *A*_1_, as previously noted for *τ*_1_. Well linked to the VO_2_ first component amplitude is the fast component Gain that evidenced a tendency to decrease with increasing intensity, in line with the decrease reported as the exercise intensity approaches the individuals' VO_2max⁡_ [47]. Comparison in the VO_2_ gain between intensities around VO_2max⁡_ (90, 100, and 110% of VO_2max⁡_) have been reported only for cycling exercise [47], being this study the first attempt to assess the VO_2_ gain during swimming exercise surrounding the VO_2max⁡_ intensity. The lack of differences seems to indicate that the 5% changes in swimming velocity were insufficient to induce an adjustment of O_2_ delivery and diffusion to the exercising muscles.

The magnitude of the VO_2_ slow component is considered to have physiological meaning only when it is ≥200 mL · min⁡^−1^ [49] (although this value is still a matter of debate), occurring at 95 and 100% of vVO_2max⁡_. At these intensities (the severe intensity domain), the attainment of a VO_2_ steady state is delayed due to the emergence of a supplementary slowly developing component of the VO_2_ response [18], corroborating the lack of differences found in *A*_2_ between 95 and 100% of vVO_2max⁡_ intensities. The *A*_2_ values found in the current study for 95 and 100% of vVO_2max⁡_ intensities are in accordance with those previously reported at 96% of vVO_2max⁡_ [7] but are higher than those presented for 100% of vVO_2max⁡_ [8, 11] and for the heavy intensity exercise domain [41, 42]. This lack of agreement could be explained by the method that was used to assess the VO_2_ slow component-fixed interval method that seems to result in lower values compared to the mathematical modeling method [39] and is considerd a simple rough estimate of this parameter [48].

At intensities higher than VO_2max⁡_ (the extreme intensity domain), the exercise duration is so short (≤2 min) that a VO_2_ slow component is not readily observed [16], confirmed at 105% of vVO_2max⁡_. The relative contribution of the VO_2_ slow component to the overall increase in VO_2_ at the end exercise was higher than those presented for heavy domain [41, 42], but being *A*_1_ and *A*_2_ dependent variables, this was an expectable outcome. Although some explanations to better understand the VO_2_ slow component phenomenon have been proposed [3], its origin in swimming is even more uncertain than in running and cycling [7]. The current study seems to indicate that possibly the type and pattern of recruitment of the available motor units were clearly modified. The lack of direct relationships between time sustained and VO_2_ slow component, as found previously [8, 17, 50], suggests that near the vVO_2max⁡_ intensity the slow component is not linked with the time sustained.

Regarding *E*_max⁡_ values, the Aer contribution was higher at 95% compared to 100 and 105% of vVO_2max⁡_ and the anaerobic (lactic and alactic) contribution evidenced the opposite trend. These relative values (percentage of the total energy spent) show similar absolute values for the Ana_lac_ and Ana_alac_ contributions (~21, 26, and 28 kJ and 29.3, 29.2, and 29.1 kJ for 95, 100, and 105% of vVO_2max⁡_, resp.) but different ones for the Aer contribution (265, 169, and 184 kJ at 95, 100, and 105% of vVO_2max⁡_, resp.). This fact can be explained by the time sustained at each intensity once the assessment of the Aer contribution was through the time integral from the VO_2_ to time curve. To date, this metabolic comparison has never been conducted at different intensities around vVO_2max⁡_. In fact, the literature regarding all energetic contributions in all sports is very scarce and, particularly in swimming, has been applied remotely [25–27]. Thus, caution must be taken when comparisons between the present results with others studies are made once the method by which the energy release was determined can have a significant influence on the calculated relative contribution of the energy systems during periods of maximal exercise [51]. At 95% of vVO_2max⁡_, the Aer contribution (~83%) was similar to the percentage proposed previously for 333 s exercise duration [26], but the Ana_lac_ (~7%) and Ana_alac_ (~10%) contributions were lower than those presented for 112 s [25] and 200 m all-out effort [27]. At 100 and 105% of vVO_2max⁡_, the Aer contributions (~74 and 59%) were similar to previous reports [25–27] but some inconsistencies were found in the remaining fractions of Ana_lac_ (~12 and 20%) and Ana_alac_ (~14 and 21%) contributions [25, 27]. The observed inverse relationship between time sustained and vVO_2max⁡_ at 100 and 105% of vVO_2max⁡_ (Figure 2) is in accordance with previous reports for similar swimming intensities [1, 5, 6, 8, 9] and could indicate a significative strenuous effort, with a more pronounced recruitment of anaerobic energy pathways (Figure 1). This fact could lead to earlier fatigue stages and, consequently, to shorter time sustained efforts. The inverse trend (greater dependency of the aerobic energy pathway) could explain the lack of an inverse relationship between time to exhaustion and vVO_2peak_ at 95% of vVO_2max⁡_.

Further studies to compare the transient VO_2_ kinetics responses and metabolic contributions whilst swimming at different velocities around maximal intensities are supported by the data from this study. However, the fact that only one transition from rest to 95, 100, and 105% of vVO_2max⁡_ intensity was done could lead to a low signal-to-noise-ratio. Consequently, this factor could have influenced the swimmers' performance and subsequent VO_2_ kinetics and, for that, should be construed as a possible limitation of the present study.

## 5. Conclusions

This study was the first attempt to examine and compare the VO_2_ kinetics and the metabolic profile during time to exhaustion exercise at different velocities around the vVO_2max⁡_ intensity. The 5% velocity variability across conditions was not sufficient to promote changes in the kinetics of the VO_2_ fast component (*τ*_1_, *A*_1_, and Gain) but resulted in differences in the kinetics of the VO_2_ slow component (*A*_2_) and the corresponding metabolic profiles. Though being well documented in cycling and running exercise, VO_2_ kinetics has received considerably less research attention in swimming, even though it is providing a noninvasive into oxidative metabolism at the muscle level. Since athletes typically train at intensities surrounding the VO_2max⁡_, understanding how subtle variations in intensity surrounding the VO_2max⁡_ impacts on oxidative metabolism and performance might have important implications for optimising high-intensity interval training.

## Figures and Tables

**Table 1:** Mean ± SD values for VO_2 max⁡_, HR_max_, R, Ve, and [La^−^]_max⁡_ obtained at the end of the incremental protocol and the time to exhaustion tests (*n* = 12).

	Incremental protocol	95% of vVO_2 max⁡_	100% of vVO_2 max⁡_	105% of vVO_2 max⁡_
VO_2 max⁡_ (L*·*min^−1^)	4.21 ± 0.61	4.36 ± 0.55	4.41 ± 0.74	4.24 ± 0.64
VO_2 max⁡_ (mL*·*kg^−1^ *·*min^−1^)	60.75 ± 5.17	61.34 ± 5.58	60.05 ± 6.10	59.78 ± 6.45
HR_max_ (beats*·*min^−1^)	180.6 ± 6.96	176.75 ± 9.63	176.91 ± 8.57	177.00 ± 8.90
R	0.93 ± 0.05	0.95 ± 0.06	1.01 ± 0.09	0.97 ± 0.06
Ve (L*·*min^−1^)	112.04 ± 23.38	116.54 ± 21.94	115.75 ± 34.25	119.27 ± 17.17
[La^−^]_max⁡_ (mmol*·*L^−1^)	7.18 ± 2.52	8.04 ± 1.70	8.86 ± 1.63	8.45 ± 1.91

VO_2 max⁡_ = maximal oxygen uptake; HR_max_ = maximal heart rate; R = respiratory quotient; Ve = ventilation; [La^−^]_max⁡_ = maximal blood lactate concentrations.

**Table 2:** Mean ± SD values for the VO_2_ kinetics responses during at the time to exhaustion tests (*n* = 12).

VO_2_ kinetics parameters	95% of vVO_2 max⁡_	100% of vVO_2 max⁡_	105% of vVO_2 max⁡_
*A*_0_ (mL*·*min^−1^)	1214.71 ± 351.79	1358.53 ± 368.71	1389.55 ± 257.32
*A*_1_ (mL*·*min^−1^)	2568.31 ± 384.22	2402.64 ± 327.82	2628.24 ± 410.31
TD_1_ (s)	11.28 ± 3.98	8.60 ± 2.49	8.05 ± 3.49
*τ*_1_ (s)	14.82 ± 4.01	18.06 ± 3.07	16.37 ± 3.81
95% confidence intervals (s)	(12.3–17.4)	(16.1–20.1)	(13.9–18.8)
DefO_2_ (L)	0.60 ± 0.12	0.77 ± 0.24	0.74 ± 0.19
Gain (mL*·*m^−1^)	32.07 ± 4.54	29.39 ± 4.72	29.84 ± 4.43
*A*_2_ (mL*·*min^−1^)	480.76 ± 247.01^b^	452.18 ± 217.04^b^	147.07 ± 60.40
TD_2_ (s)	106.29 ± 28.67^a,b^	59.99 ± 12.50	69.07 ± 5.70
*τ*_2_ (s)	120.23 ± 31.77^b^	121.12 ± 31.71^b^	61.46 ± 27.29
% *A*_2_	15.81 ± 7.87^b^	16.36 ± 6.04^b^	5.32 ± 1.99

*A*_0_ = VO_2_ just before the beginning of exercise; *A*_1_, TD_1_, *τ*_1_, DefO_2_, and Gain = fast component amplitude, time delay, time constant, O_2_ deficit and gain, respectively; *A*_2_, TD_2_, *τ*_2_, and % *A*_2_ = slow component amplitude, time delay, time constant, and relative contribution of slow component in relation to the end exercise VO_2_ of that bout, respectively. Differences between intensities are identified by a and b (100 and 105% of vVO_2 max⁡_) (*P* ≤ 0.05).
